# Linear growth trajectories in the first two years of life predict attained linear growth and stunting at five years: Results from the MAL-ED multi-country birth cohort study

**DOI:** 10.1371/journal.pone.0329596

**Published:** 2026-04-06

**Authors:** Md. Ashraful Alam, S. M. Tafsir Hasan, Amena Al Nishan, Mustafa Mahfuz, Margaret N. Kosek, Aldo A. M. Lima, Bruna L. L. Maciel, Tahmeed Ahmed

**Affiliations:** 1 Nutrition Research Division, International Centre for Diarrhoeal Disease Research, Bangladesh (icddr, b), Dhaka, Bangladesh; 2 Poche Centre for Indigenous Health, The University of Queensland, Brisbane, Queensland, Australia; 3 Division of Infectious Diseases and International Health, School of Medicine, University of Virginia, Charlottesville, Virginia, United States of America; 4 Universidade Federal do Ceará, Fortaleza, Brazil; 5 Universidade Federal do Rio Grande do Norte, Natal, Brazil; 6 Office of Executive Director, International Centre for Diarrhoeal Disease Research, Bangladesh, (icddr, b), Dhaka, Bangladesh; Flinders University, AUSTRALIA

## Abstract

**Background:**

Linear growth faltering and stunting are associated with increased childhood mortality and morbidity, as well as impaired physical growth and cognitive development.

**Objectives:**

We aimed to identify groups of children with distinct linear growth trajectories during their first two years of life and to determine whether these early-life trajectories were associated with attained linear growth and stunting at five years of age.

**Methods:**

We used data from the MAL-ED birth cohort study for this analysis. Latent class growth modeling (LCGM) was applied to identify distinct classes of children who followed similar trajectories of length-for-age z-score (LAZ) from 0 to 24 months of age. Mixed-effects linear and logistic regression models were used to examine the associations between LCGM-derived trajectories and height-for-age z-score (HAZ) and stunting at age 60 months, respectively, with study site included as a random effect.

**Results:**

We identified five LAZ trajectories among 1469 children aged 0–24 months, designated as follows: Class 1: severely attenuated linear growth (9%); Class 2: moderately attenuated linear growth (25%); Class 3: mildly attenuated linear growth (34%); Class 4: stable linear growth (25%); Class 5: improved linear growth (7%). In adjusted models, LAZ trajectories during the first two years of life were significantly associated with HAZ and stunting at five years. Compared with the stable linear growth class, children in the improved linear growth class had a predicted 0.86 higher HAZ at age five (95% CI: 0.67, 1.04), whereas those in the severely attenuated linear growth class had substantially lower HAZ at age five (β = −2.10; 95% CI: −2.26, −1.95). Similar associations were observed for stunting at five years.

**Conclusions:**

Linear growth trajectories during the first two years of life are critical predictors of attained linear growth and stunting at five years. Greater emphasis should be placed on improving early-life linear growth through community-based interventions.

## Introduction

Linear growth faltering in children remains a major global health challenge, with a staggering 148.1 million stunted children under the age of 5 years in low and middle-income countries (LMICs) [1]. Evidence suggests that early-life stunting is associated with an increased risk of childhood mortality [2], infectious diseases such as diarrhea, pneumonia, and measles [3,4], impaired growth and cognitive development [5–8], as well as poor adult economic outcomes [9]. Furthermore, recent studies have demonstrated that impaired linear growth in early life is associated with subsequent deficits in later childhood, with infrequent reversal of stunting [10,11].

While several nutritional interventions have shown potential in addressing childhood stunting, achieving widespread success remains elusive [12]. Moreover, growth during the first thousand days is influenced by several factors, including environmental exposures, infectious diseases, and malnutrition, and is not homogeneous in pattern [13,14]. As children follow heterogeneous growth patterns [15], characterizing childhood growth trajectories and tailoring interventions to distinct groups has become essential. Novel, trajectory-informed strategies may strengthen the effectiveness of interventions aimed at preventing childhood stunting.

Group-based trajectory models can classify individuals with similar features, characteristics, and attributes into discrete, unambiguous subgroups [16–18] and have become increasingly useful in understanding patterns of child growth and development. While prior research has established a link between early growth patterns during the first 24 months of life and subsequent growth, these trajectories have not been adequately explored using the latent class growth modeling (LCGM) technique. LCGM is a unique approach because it models population-level developmental heterogeneity as distinct, interpretable trajectory groups rather than as individual-level random variation [19,20]. Unlike traditional growth models, LCGM identifies groups that cannot be directly observed [20]. Although some studies have explored trends in linear growth in low-income settings [21,22], comprehensive data from LMICs regarding the presence of distinct group-based trajectories of linear growth among young children remain limited. Few studies have applied group-based or latent trajectory approaches to identify heterogeneous patterns, such as early growth faltering, catch-up growth, or persistently adequate growth, within the same population. This knowledge gap limits our understanding of growth heterogeneity in LMICs and constrains the ability to identify critical windows for intervention and to design targeted strategies for children most at risk of sustained linear growth faltering. It also remains unclear to what extent early-life linear growth trajectories influence and set the course for linear growth attained later in childhood.

This study aimed to identify groups of children with distinct linear growth trajectories during the first two years of life in a birth cohort from seven LMICs. It also assessed the association between these early-life linear growth trajectories and attained linear growth and stunting at 60 months of age.

## Methods

### Ethics statement

The study was conducted in accordance with the guidelines of the Declaration of Helsinki. For the parent study, ethical approval was obtained from the Institutional Review Boards at each of the participating research sites and at the University of Virginia School of Medicine (Charlottesville, USA). The current study (Protocol ID: PR-2008–020) was approved by the Research Review Committee and the Ethical Review Committee (Institutional Review Board) of icddr,b. Written informed consent was obtained from the legal guardians of all participants.

### Study setting and participants

The MAL-ED study design, site descriptions, sample size calculation, and methods have been described previously in detail [23]. Briefly, the MAL-ED study is a multi-country, prospective, community-based longitudinal birth cohort study conducted at eight locations in LMICs [23]. Data were collected between November 2009 and February 2017 in Fortaleza, Brazil (BRF); Dhaka, Bangladesh (BGD); Vellore, India (INV); Loreto, Peru (PEL); Bhaktapur, Nepal (NEB); Naushero Feroze, Pakistan (PKN); Haydom, Tanzania (TZH) and Venda, South Africa (SAV). Initially, each site planned to enroll 200 children at birth and follow them through 24 months of age. Subsequently, through an amendment to the original protocol, follow-up was extended to 60 months of age. Eligible newborns were fewer than 17 days old, born singletons with a birth weight >1500 g, free of significant illnesses, born to mothers aged at least 16 years old, and whose families planned to stay in the community for at least 6 months. Pakistan was excluded from the present analyses due to bias in length measurements in a subgroup of participants.

### Anthropometry, dietary intake, and socioeconomic status

From birth to 60 months of age, trained field staff measured children’s recumbent length (using a Seca 417 Infantometer) or standing height (using a Seca 213 portable stadiometer) monthly to the nearest 0.1 cm following standard procedures. We derived length/height-for-age z scores using the World Health Organization (WHO) growth standards [24]. Stunting at age 60 months was defined as a height-for-age z score < −2.

Trained field workers assessed dietary intake at 60 months using quantitative 24-hour recalls [25]. The dietary intake data underwent conversion to estimate energy, macronutrient, and micronutrient intakes from non-breast milk foods, employing site-specific food composition databases developed within MAL-ED.

The MAL-ED study developed a composite socioeconomic status (SES) score that could be compared across study sites. The SES construct combines improved access to water and sanitation, maternal education, household assets, and average monthly household income into a composite index (WAMI) ranging from 0 to 1 [26].

### Statistical analysis

From 0 to 24 months of age, LCGM was used to identify distinct classes or clusters of children who followed similar trajectories of length-for-age z score (LAZ). LCGM is a semi-parametric finite mixture modeling technique that uses maximum likelihood estimation to analyze longitudinal data and identify distinct, meaningful groups of individuals who share similar progression over time for a particular variable. LCGM relaxes the assumption that all individuals originate from a single homogeneous population, allowing growth parameters to vary across unobserved subpopulations. However, within each trajectory group, the slope and intercept are assumed to be constant [27,28].

For LAZ, distinct trajectory models were constructed. LCGM requires at least three measurement time points per individual to estimate trajectories reliably [29]; a total of 25 measurement time points were available for the present analysis. This analysis included only children with both 0- and 24-month LAZ data.

We developed and compared models with 1–5 trajectories. LAZ was modeled using a censored normal distribution. Models of increasing complexity were fitted, starting with a single trajectory, and the optimal number of trajectories was determined based on model fit. Linear, quadratic, cubic, and quartic functions of time (age in months) were examined to identify the best-fitting trajectory shapes. Models that produced trajectories with insufficient cluster size (<5% of the study population) were not considered.

Model selection was based on the Bayesian information criteria (BIC), log Bayes factor, statistical significance of higher-order polynomial terms, overlap of 95% confidence intervals (95% CI) between trajectories, and the proportion of participants in each trajectory group. The model with the smallest absolute BIC value was considered to have the best fit [30]. After selecting the final model, posterior probabilities of group membership were estimated for each individual. Children were assigned to trajectory groups using the maximum posterior probability criterion [31].

To determine the final model’s goodness of fit, we examined whether the average posterior probability of assignment for each subgroup was greater than 0.8, whether the odds of correct classification were greater than 5, and whether the modeled group probabilities were in good agreement with the proportions of group assignments [29]. Results were reported following *the GRoLTS-Checklist: Guidelines for Reporting on Latent Trajectory Studies* [32].

Linear mixed-effects models were used to investigate the association between HAZ at 60 months and the LAZ trajectories identified by LCGM, including a random intercept for the study site to account for clustering. This approach allows appropriate estimation of variability both within and between sites [33]. Additionally, mixed-effects logistic regression models were applied to assess the association between stunting at 60 months and the LAZ trajectory groups, also including a random intercept for site. Due to the small number of stunted children, trajectory classes 3, 4, and 5 were combined for the analysis. Multivariable models were adjusted for covariates measured at 60 months included total protein intake (g/day), total fat intake (g/day), total carbohydrate intake (g/day), socioeconomic status (WAMI score), and child sex. The strength of association was expressed as mean difference (β) and odds ratio (OR) with 95% CI for the linear and logit models, respectively.

LCGM analyses were conducted using the “traj plugin” in Stata [34], the Stata equivalent of the widely used “proc traj” procedure in SAS [30]. The R packages “lcmm” and “ggplot2” were used to visualize trajectory outputs. All other statistical analyses were performed using Stata version 15.0 (StataCorp, College Station, TX, USA).

## Results

**Fig 1:**
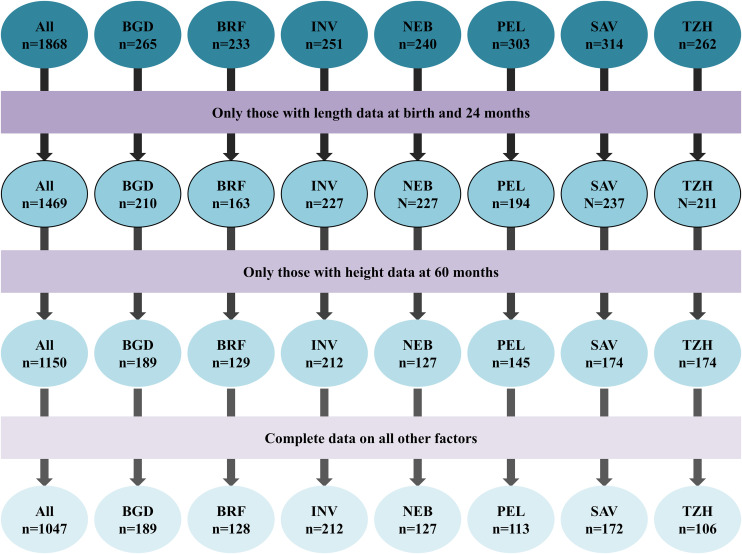
Flow diagram of participant inclusion and retention in the analysis from the MAL-ED birth cohort study. Sites: BGD: Bangladesh – Dhaka; INV: India – Vellore; NEB: Nepal – Bhaktapur; BRF: Brazil – Fortaleza; PEL: Loreto, Peru; SAV: South Africa – Venda; TZH: Tanzania – Haydom.

The MAL-ED study enrolled 1,868 children from seven countries (Bangladesh, India, Nepal, Brazil, Peru, South Africa, and Tanzania). Of these, 1469 children had length data at birth and 24 months, and 1047 children had complete data on all other variables (Fig 1).

Of the children, 49% were girls. The average length-for-age z score at birth ranged from −1.02 in INV and TZH to −0.71 in SAV. Overall, median protein consumption was 37.7 g/d with the lowest median protein intake in NEB (30.4 g/d) and the highest median protein intake in BRF (54.0 g/d) at 60 months of age. The seven locations had different median fat intakes, ranging from 21.3 g/d in TZH to 45.4 g/d in BRF. The carbohydrate intakes in the seven areas varied, ranging from 171 g/d in NEB to 318 g/d in TZH (Table 1).

**Table 1:** Study population characteristics.

Characteristics	Overall	BGD	BRF	INV	NEB	PEL	SAV	TZH
**Birth history**								
Number of children	1469	210	163	227	227	194	237	211
Girls, n (%)	715 (48.7%)	102 (48.6%)	76 (46.6%)	122 (53.7%)	105 (46.3%)	89 (45.9%)	117 (49.4%)	104 (49.3%)
Length-for-age z score, Mean (SD)	−0.88 (1.05)	−0.97 (1.01)	−0.82 (1.13)	−1.02 (1.05)	−0.72 (1.03)	−0.95 (0.96)	−0.71 (0.99)	−1.02 (1.15)
**Age 60 months**								
Number of children	1047	189	128	212	127	113	172	106
Daily protein intake in grams, Median (IQR)	37.7 [29.6, 48.7]	30.9 [27.5, 35.5]	54.0 [44.6, 61.9]	37.3 [29.8, 44.3]	30.4 [24.6, 35.9]	33.7 [26.7, 41.9]	48.2 [37.1, 58.5]	44.0 [37.3, 54.2]
Daily fat intake in grams, Median (IQR)	34.8 [25.9, 45.5]	34.4 [28.1, 40.1]	45.4 [38.0, 54.4]	42.5 [32.1, 53.1]	33.9 [26.0, 42.5]	27.6 [18.8, 36.5]	32.6 [25.6, 43.0]	21.3 [15.6, 27.7]
Daily carbohydrate intake in grams, Median (IQR)	212 [170, 267]	173 [156, 196]	208 [185, 240]	253 [216, 294]	171 [143, 202]	221 [180, 277]	229 [172, 279]	318 [257, 417]
WAMI index, Mean (SD)	0.62 (0.20)	0.61 (0.13)	0.83 (0.08)	0.58 (0.15)	0.73 (0.15)	0.55 (0.13)	0.74 (0.13)	0.25 (0.12)
Length-for-age z score, Mean (SD)	−1.28 (1.05)	−1.60 (0.89)	−0.13 (0.99)	−1.55 (0.92)	−1.25 (0.89)	−1.38 (0.87)	−0.95 (0.93)	−2.07 (0.91)

Sites: BGD: Bangladesh – Dhaka; INV: India – Vellore; NEB: Nepal – Bhaktapur; BRF: Brazil – Fortaleza; PEL: Loreto, Peru; SAV: South Africa – Venda; TZH: Tanzania – Haydom.

### Results of LCGM

**Fig 2:**
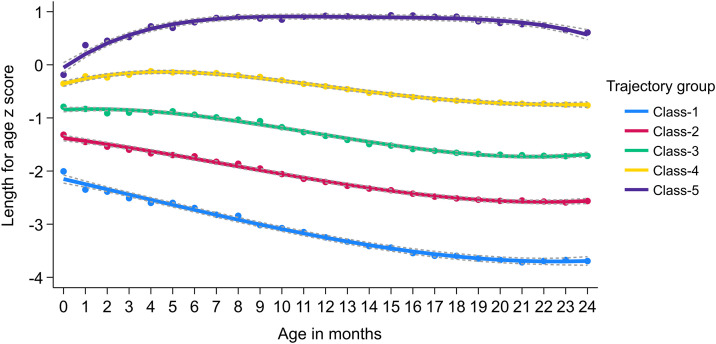
LCGM-derived latent trajectories of length-for-age z-score (LAZ) from 0-24 months with 95% confidence intervals. Trajectory group: Class 1: Severely attenuated; Class 2: Moderately attenuated; Class 3: Mildly attenuated; Class 4: Stable; Class 5: Improved.

The population distribution of LAZ progression was best described by a five-class model, with cubic terms for the first three trajectories and quartic terms for the remaining two. LAZ trajectory class 1, termed “severely attenuated linear growth,” represented 9% of the children in the sample. The group was born with a mean LAZ of −2.15 and experienced a marked decline through 24 months (mean LAZ = −2.73, −3.25, −3.60, and −3.69 at 6, 12, 18, and 24 months, respectively). Trajectory class 2, labeled “moderately attenuated linear growth,” accounted for 25% of participants. These children had a mean LAZ of −1.37 at birth, which progressively declined to −1.76, −2.19, −2.50, and −2.56 at 6, 12, 18, and 24 months, respectively. Trajectory class 3, termed “mildly attenuated linear growth,” comprised 34% of participants. This group had a mean LAZ of −0.84 at birth and showed a gradual decline through 24 months. Trajectory class 4, termed “stable linear growth,” included 25% of participants. Their mean LAZ increased slightly during the first five months of life and then gradually declined through 24 months. Trajectory class 5, labeled “improved linear growth,” represented 7% of children. This group was born with a mean LAZ of −0.05, demonstrated substantial improvement up to 12 months of age, and then showed a modest decline through 24 months (mean LAZ = 0.82, 0.91, 0.87, and 0.57 at 6, 12, 18, and 24 months, respectively) (Fig 2) (Table 2).

**Table 2:** Estimated mean length-for-age z-score (LAZ) at different ages by trajectory group, derived using LCGM, with 95% confidence intervals.

Age in months	Length-for-age z-score, mean (95% CI)				
	Class 1	Class 2	Class 3	Class 4	Class 5
**0**	−2.15 (−2.23, −2.07)	−1.38 (−1.43, −1.33)	−0.84 (−0.88, −0.80)	−0.35 (−0.41, −0.30)	−0.05 (−0.14, 0.04)
**1**	−2.25 (−2.30, −2.19)	−1.43 (−1.48, −1.39)	−0.83 (−0.86, −0.80)	−0.26 (−0.30, −0.23)	0.20 (0.14, 0.26)
**2**	−2.34 (−2.39, −2.30)	−1.49 (−1.53, −1.46)	−0.84 (−0.86, −0.81)	−0.20 (−0.23, −0.17)	0.40 (0.36, 0.45)
**3**	−2.44 (−2.48, −2.40)	−1.56 (−1.59, −1.52)	−0.85 (−0.88, −0.82)	−0.15 (−0.18, −0.12)	0.56 (0.51, 0.61)
**4**	−2.54 (−2.58, −2.50)	−1.63 (−1.66, −1.59)	−0.88 (−0.90, −0.85)	−0.13 (−0.16, −0.11)	0.68 (0.63, 0.73)
**5**	−2.63 (−2.68, −2.59)	−1.70 (−1.73, −1.66)	−0.91 (−0.94, −0.88)	−0.13 (−0.16, −0.10)	0.76 (0.72, 0.81)
**6**	−2.73 (−2.77, −2.69)	−1.77 (−1.80, −1.73)	−0.96 (−0.98, −0.93)	−0.15 (−0.17, −0.12)	0.82 (0.78, 0.87)
**7**	−2.82 (−2.87, −2.78)	−1.84 (−1.87, −1.81)	−1.01 (−1.03, −0.98)	−0.17 (−0.20, −0.15)	0.86 (0.82, 0.91)
**8**	−2.92 (−2.96, −2.87)	−1.91 (−1.94, −1.88)	−1.06 (−1.09, −1.03)	−0.21 (−0.23, −0.18)	0.89 (0.85, 0.93)
**9**	−3.01 (−3.05, −2.96)	−1.98 (−2.02, −1.95)	−1.12 (−1.15, −1.10)	−0.25 (−0.28, −0.23)	0.90 (0.86, 0.94)
**10**	−3.09 (−3.13, −3.05)	−2.06 (−2.09, −2.02)	−1.19 (−1.21, −1.16)	−0.30 (−0.33, −0.27)	0.91 (0.87, 0.95)
**11**	−3.17 (−3.22, −3.13)	−2.13 (−2.16, −2.10)	−1.25 (−1.28, −1.23)	−0.35 (−0.38, −0.32)	0.91 (0.86, 0.95)
**12**	−3.25 (−3.30, −3.21)	−2.19 (−2.22, −2.16)	−1.32 (−1.35, −1.29)	−0.40 (−0.43, −0.38)	0.91 (0.86, 0.95)
**13**	−3.33 (−3.37, −3.28)	−2.26 (−2.29, −2.23)	−1.39 (−1.41, −1.36)	−0.46 (−0.48, −0.43)	0.90 (0.86, 0.95)
**14**	−3.39 (−3.44, −3.35)	−2.32 (−2.35, −2.29)	−1.45 (−1.47, −1.42)	−0.51 (−0.53, −0.48)	0.90 (0.85, 0.94)
**15**	−3.46 (−3.51, −3.41)	−2.37 (−2.40, −2.34)	−1.51 (−1.54, −1.48)	−0.56 (−0.58, −0.53)	0.89 (0.85, 0.94)
**16**	−3.51 (−3.57, −3.46)	−2.42 (−2.45, −2.39)	−1.56 (−1.59, −1.54)	−0.60 (−0.63, −0.57)	0.89 (0.84, 0.93)
**17**	−3.56 (−3.62, −3.51)	−2.47 (−2.5, −2.44)	−1.61 (−1.64, −1.59)	−0.64 (−0.67, −0.61)	0.88 (0.83, 0.93)
**18**	−3.61 (−3.66, −3.55)	−2.51 (−2.54, −2.48)	−1.66 (−1.68, −1.63)	−0.67 (−0.70, −0.64)	0.87 (0.82, 0.92)
**19**	−3.64 (−3.70, −3.59)	−2.54 (−2.57, −2.51)	−1.69 (−1.72, −1.66)	−0.70 (−0.73, −0.67)	0.86 (0.80, 0.91)
**20**	−3.67 (−3.72, −3.62)	−2.56 (−2.59, −2.53)	−1.72 (−1.74, −1.69)	−0.72 (−0.75, −0.69)	0.83 (0.78, 0.89)
**21**	−3.69 (−3.74, −3.64)	−2.58 (−2.61, −2.54)	−1.73 (−1.75, −1.70)	−0.74 (−0.77, −0.71)	0.80 (0.74, 0.85)
**22**	−3.70 (−3.76, −3.64)	−2.58 (−2.62, −2.55)	−1.73 (−1.76, −1.70)	−0.75 (−0.78, −0.72)	0.74 (0.69, 0.80)
**23**	−3.70 (−3.77, −3.64)	−2.58 (−2.62, −2.54)	−1.72 (−1.75, −1.68)	−0.75 (−0.79, −0.71)	0.67 (0.61, 0.73)
**24**	−3.69 (−3.77, −3.61)	−2.56 (−2.61, −2.51)	−1.69 (−1.73, −1.65)	−0.75 (−0.80, −0.70)	0.57 (0.47, 0.66)

Class 1: Severely attenuated; Class 2: Moderately attenuated; Class 3: Mildly attenuated; Class 4: Stable; Class 5: Improved.

**Fig 3:**
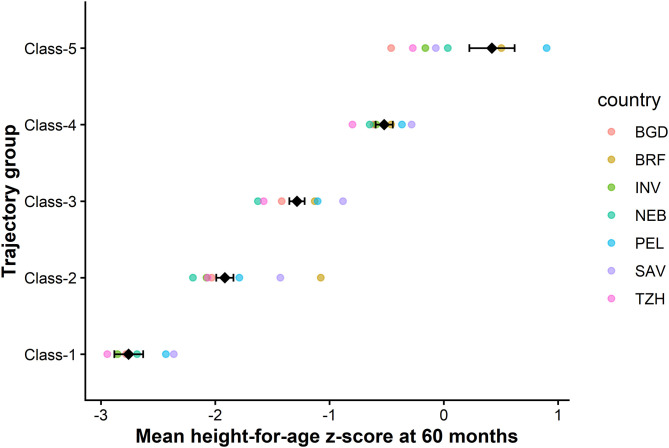
Mean height-for-age z-score at 60 months by trajectory group across the seven study sites. Trajectory group: Class 1: Severely attenuated; Class 2: Moderately attenuated; Class 3: Mildly attenuated; Class 4: Stable; Class 5: Improved.

Across all trajectories, the average posterior probability of group membership exceeded 0.90, the odds of correct classification were greater than 50, and modeled group probabilities were in good agreement with the observed proportions of assigned group membership (Fig 3) (Table 3).

**Table 3:** Fit statistics for the final LCGM model of length-for-age z-score (LAZ).

LAZ trajectory group	n	Average posterior probability	Odds of correct classification	Estimated group probability	Proportion of group assignment
Class 1: Severely attenuated	132	0.98	523.93	0.09	0.09
Class 2: Moderately attenuated	373	0.98	139.93	0.26	0.25
Class 3: Mildly attenuated	495	0.97	71.38	0.33	0.34
Class 4: Stable	363	0.98	176.25	0.25	0.25
Class 5: Improved	106	0.99	2706.68	0.07	0.07

### Effect of LAZ trajectories on HAZ at 5 years

LAZ trajectories during the first two years of life were associated with HAZ at five years of age (Table 4).

**Table 4:** Association of LAZ trajectories during the first two years of life with height-for-age z-score (HAZ) at age 60 months (n = 1047).

LAZ trajectory group	Height-for-age z-score (HAZ)			
	Unadjusted		Adjusted^1^	
	β (95% CI)	p-value	β (95% CI)	p-value
Class 1: Severely attenuated	−2.14 (−2.30, −1.98)	<0.001	−2.10 (−2.26, −1.94)	<0.001
Class 2: Moderately attenuated	−1.37 (−1.49, −1.26)	<0.001	−1.34 (−1.45, −1.23)	<0.001
Class 3: Mildly attenuated	−0.73 (−0.84, −0.62)	<0.001	−0.71 (−0.81, −0.60)	<0.001
Class 4: Stable	Reference		Reference	
Class 5: Improved	0.86 (0.67, 1.05)	<0.001	0.86 (0.67, 1.04)	<0.001

^1^Adjusted for child sex, daily average fat, protein and carbohydrate intake and well as socio-economic status (WAMI).

After adjusting for potential confounders, the improved linear growth class had a predicted 0.86 higher HAZ at five years of age (95% CI: 0.67, 1.04), whereas the severely, moderately, and mildly attenuated linear growth classes had significantly lower HAZ at five years compared with the stable linear growth class (severely: β = −2.10; 95% CI: −2.26, −1.95; moderately: β = −1.34; 95% CI: −1.45, −1.22; mildly: β = −0.70; 95% CI: −0.81, −0.59).

### Effect of LAZ trajectories on stunting at 5 years

LAZ trajectories during the first two years of life were significantly associated with stunting at five years of age (Table 5).

**Table 5:** Association of LAZ trajectories during the first two years of life with stunting at age 60 months (n = 1047).

LAZ trajectory group	Stunting			
	Unadjusted		Adjusted^1^	
	OR (95% CI)	p-value	OR (95% CI)	p-value
Severely attenuated (Class 1)	142.08 (67.24, 300.20)	<0.001	132.78 (61.58, 286.52)	<0.001
Moderately attenuated (Class 2)	16.95 (10.91, 26.35)	<0.001	16.00 (10.28, 24.91)	<0.001
Mildly attenuated or Stable or Improved (Class 3 or 4 or 5)	Reference		Reference	

^1^Adjusted for child sex, daily average fat, protein and carbohydrate intake and well as socio-economic status (WAMI).

After adjusting for potential confounders, the severely attenuated linear growth class (OR 132.78; 95% CI: 61.58, 286.52) and the moderately attenuated linear growth class (OR 16.00; 95% CI: 10.28, 24.91) had higher odds of stunting at the age of five years compared with the other three trajectory classes.

## Discussion

To our knowledge, this is the first study to identify discrete linear growth trajectories during the first two years of life and to examine their association with attained linear growth and stunting in later childhood in a multi-country birth cohort from low-income settings. We identified five distinct linear growth trajectories from birth to 24 months using latent class growth modeling. Namirembe et al. identified four distinct linear growth trajectories from birth to 12 months among children in rural Uganda [35]. Similarly, a longitudinal birth cohort study of HIV-unexposed children in Zimbabwe identified four distinct linear growth trajectories from birth through two years of age by clustering infants with similar growth patterns [36].

Of the five trajectory classes identified in our study, the “severely attenuated linear growth” group had the lowest mean LAZ at birth (−2.15), already within the stunted range, and declined further to −3.69 by 24 months, reaching the severely stunted range. In contrast, the “improved linear growth” group started as non-stunted (mean LAZ −0.05 at birth), improved through 12 months, and showed only slight tapering by 24 months (mean LAZ 0.57). Both groups represented relatively small proportions of participants (9% and 7%, respectively). The largest proportion of children (34%) belonged to the “mildly attenuated linear growth” trajectory. Although this group started in the non-stunted range (mean LAZ −0.84 at birth), their mean LAZ gradually declined to −1.69 at 24 months, placing them in the at-risk-of-stunting range. The remaining two trajectories each comprised 25% of the cohort. The “moderately attenuated linear growth” group started in the at-risk-of-stunting range (mean LAZ −1.38 at birth), declined steadily, and reached the stunted range by 24 months (mean LAZ −2.56). In contrast, children in the “stable linear growth” trajectory maintained a relatively stable growth and remained within the non-stunted range throughout the first 24 months.

Among the five trajectory classes, the two groups with the lowest mean LAZ at birth, namely the “severely attenuated linear growth” and “moderately attenuated linear growth”, demonstrated markedly elevated odds of stunting at five years of age compared with the other three groups. Complementing this finding, Krebs et al. reported that deficits in birth length were strong predictors of attained LAZ and stunting at the age of two years across sites in the Democratic Republic of Congo, Guatemala, India, and Pakistan [37]. Mild and moderate stunting at birth were associated with a 0.5–1.0 lower mean LAZ and a 40–60% increased risk of stunting at two years [37].

Our findings further suggest a graded association between early-life linear growth trajectories and attained HAZ at five years of age. Compared with the stable linear growth class, the mildly, moderately, and severely attenuated linear growth trajectories were progressively associated with lower HAZ at five years, demonstrating a dose-response pattern. Conversely, the improved linear growth class was associated with higher HAZ at five years. Although the biological mechanism underlying these graded effects remains unclear, accumulating evidence indicates that early life growth faltering has long-term and potentially lifelong consequences for health outcomes [38,39]. Nutrition during the first 1,000 days of life plays a critical role in shaping growth and development across the life course [40,41].

Roth et al., analyzing 179 Demographic and Health Surveys from 64 LMICs using Monte Carlo simulations, presented an important population-level perspective [42]. They demonstrated that as mean HAZ declined from birth to three years, both tails of the distribution (5th and 95th percentiles) shifted downward, resulting in a nearly symmetrical narrowing of the overall distribution [42]. This age-related distributional shift suggests that children across the entire HAZ spectrum experience postnatal linear growth faltering, supporting the view that postnatal growth faltering in LMICs should be considered a whole-population condition [42]. While Roth et al. demonstrated a population-level downward shift in the HAZ distribution in LMICs, our findings extend this perspective by identifying distinct longitudinal growth trajectories within that broader pattern of faltering.

WHO nutritional status categories (normal, stunted, severely stunted) provide cross-sectional classifications based on length/height-for-age at a given time point. In contrast, the trajectory classes identified through LCGM reflect longitudinal growth patterns over time. The practical implication of identifying five trajectory classes is that children with similar HAZ at 24 months may have followed different growth pathways (e.g., persistently faltering versus improving), which may carry different implications for growth at 60 months. Although some trajectory classes, particularly the severely and moderately attenuated groups, may overlap with children classified as stunted under WHO standards at specific ages, the two approaches are conceptually distinct. While WHO standards classify growth status relative to an external reference population at a single time point, our trajectory-based classification captured internally derived, dynamic growth processes across early life and therefore provided additional insight beyond conventional categories.

The distinct patterns identified in this study highlight the importance of tracking early-life growth trajectories and implementing group-specific strategies to prevent growth faltering. Interventions based solely on population averages may overlook children at the highest risk. Consideration of the timing of growth faltering, recovery patterns, and contextual determinants is essential when developing targeted strategies to improve child growth and health. We recommend the development and implementation of trajectory-informed strategies and policies that account for children’s specific growth patterns alongside nutritional status, WASH conditions, environment, and socioeconomic context, consistent with broader policy recommendations [43,44].

This study has several limitations. Although we characterized early-life linear growth trajectories and demonstrated their association with attained linear growth in later childhood, we did not investigate determinants of trajectory membership. Important early-life factors such as parental height, maternal nutritional status, gestational weight gain, placental stress or insufficiency, length of gestation, and size at birth were not fully accounted for due to data limitations. Catch-up growth among infants born small for gestational age and catch-down growth among those born large for gestational age were also not examined because gestational age–specific birth size data were limited. In addition, environmental exposures, including infections, morbidity, and nutritional intake between 25 and 59 months, were not comprehensively captured. However, we adjusted for the WAMI index, a composite indicator reflecting socioeconomic and environmental conditions (including SES and WASH components), as well as dietary intake at 60 months. Future research should examine the determinants and causal pathways underlying distinct growth trajectories to better inform preventive strategies.

## Conclusion

This study identified five distinct linear growth trajectories during the first two years of life among children from seven LMICs, ranging from severely attenuated to improved growth patterns. These early-life trajectories were strongly associated with attained linear growth and stunting at five years of age, demonstrating a graded relationship across trajectory classes. While current public health strategies rely primarily on WHO-defined thresholds of stunting, trajectory-based classification may offer complementary insights by capturing longitudinal growth dynamics and identifying children at risk of persistent growth faltering before they meet conventional criteria. Integrating longitudinal growth patterns with existing frameworks may strengthen early identification and inform timely, context-specific interventions aimed at preventing long-term growth deficits.

## Supporting information

S1 Table Association of LAZ trajectories during the first two years of life with height-for-age z-score (HAZ) at age 60 months (n = 1047): full adjusted model. (DOCX)

S2 Table Association of LAZ trajectories during the first two years of life with stunting at age 60 months (n = 1047): full adjusted model. (DOCX)

S1 Appendix STROBE checklist. (DOCX)

S2 Appendix Inclusivity in global research questionnaire. (DOCX)

## References

[1] World Health Organisation. Levels and trends in child malnutrition: UNICEF/WHO/World Bank Group joint child malnutrition estimates: key findings of the 2023 edition. 2023.

[2] Thurstans S, Wrottesley SV, Fenn B, Khara T, Bahwere P, Berkley JA, et al. Anthropometric deficits and the associated risk of death by age and sex in children aged 6-59 months: A meta-analysis. Matern Child Nutr. 2023;19(1):e13431. doi: 10.1111/mcn.13431 36164997 PMC9749608

[3] Olofin I, McDonald CM, Ezzati M, Flaxman S, Black RE, Fawzi WW, et al. Associations of suboptimal growth with all-cause and cause-specific mortality in children under five years: A pooled analysis of ten prospective studies. PLoS One. 2013;8(5):e64636. doi: 10.1371/journal.pone.0064636 23734210 PMC3667136

[4] Gausman J, Kim R, Li Z, Tu L, Rajpal S, Joe W. Comparison of child undernutrition anthropometric indicators across 56 low- and middle-income countries. JAMA Network Open. 2022;5(3):e221223-e. doi: 10.1001/jamanetworkopen.2022.1223 PMC8917428 35275168

[5] de Onis M, Branca F. Childhood stunting: A global perspective. Matern Child Nutr. 2016;12 Suppl 1(Suppl 1):12–26. doi: 10.1111/mcn.12231 27187907 PMC5084763

[6] Prendergast AJ, Humphrey JH. The stunting syndrome in developing countries. Paediatr Int Child Health. 2014;34(4):250–65. doi: 10.1179/2046905514Y.0000000158 25310000 PMC4232245

[7] Alam MA, Richard SA, Fahim SM, Mahfuz M, Nahar B, Das S, et al. Impact of early-onset persistent stunting on cognitive development at 5 years of age: Results from a multi-country cohort study. PLoS One. 2020;15(1):e0227839. doi: 10.1371/journal.pone.0227839 31978156 PMC6980491

[8] Grantham-McGregor S, Cheung YB, Cueto S, Glewwe P, Richter L, Strupp B, et al. Developmental potential in the first 5 years for children in developing countries. Lancet. 2007;369(9555):60–70. doi: 10.1016/S0140-6736(07)60032-4 17208643 PMC2270351

[9] McGovern ME, Krishna A, Aguayo VM, Subramanian SV. A review of the evidence linking child stunting to economic outcomes. Int J Epidemiol. 2017;46(4):1171–91. doi: 10.1093/ije/dyx017 28379434 PMC5837457

[10] Benjamin-Chung J, Mertens A, Colford Jr JM, Hubbard AE, van der Laan MJ, Coyle J. Early childhood linear growth faltering in low-and middle-income countries. MedRxiv. 2020. doi: 2020.06.09.20127001 10.1038/s41586-023-06418-5 PMC10511325 37704719

[11] Mertens A, Benjamin-Chung J, Colford Jr JM, Coyle J, van der Laan MJ, Hubbard AE, et al. Causes and consequences of child growth failure in low-and middle-income countries. MedRxiv. 2020. doi: 10.1101/2020.06.09.20127100

[12] Bhutta ZA, Ahmed T, Black RE, Cousens S, Dewey K, Giugliani E, et al. What works? Interventions for maternal and child undernutrition and survival. Lancet. 2008;371(9610):417–40. doi: 10.1016/S0140-6736(07)61693-6 18206226

[13] Millward DJ. Nutrition, infection and stunting: the roles of deficiencies of individual nutrients and foods, and of inflammation, as determinants of reduced linear growth of children. Nutr Res Rev. 2017;30(1):50–72. doi: 10.1017/S0954422416000238 28112064

[14] Touwslager RNH, Gielen M, Derom C, Mulder ALM, Gerver W-JM, Zimmermann LJ, et al. Determinants of infant growth in four age windows: A twin study. J Pediatr. 2011;158(4):566-572.e2. doi: 10.1016/j.jpeds.2010.10.005 21147487

[15] Regnault N, Gillman MW. Importance of characterizing growth trajectories. Ann Nutr Metab. 2014;65(2–3):110–3. doi: 10.1159/000365893 25413648 PMC4904831

[16] Nagin DS, Odgers CL. Group-based trajectory modeling in developmental science. Handbook of developmental research methods. New York, NY, US: The Guilford Press. 2012. 464–80.

[17] Martins C, Belsky J, Marques S, Baptista J, Silva J, Mesquita AR, et al. Diverse physical growth trajectories in institutionalized Portuguese children below age 3: Relation to child, family, and institutional factors. J Pediatr Psychol. 2013;38(4):438–48. doi: 10.1093/jpepsy/jss129 23262223

[18] Giles LC, Whitrow MJ, Davies MJ, Davies CE, Rumbold AR, Moore VM. Growth trajectories in early childhood, their relationship with antenatal and postnatal factors, and development of obesity by age 9 years: results from an Australian birth cohort study. Int J Obes (Lond). 2015;39(7):1049–56. doi: 10.1038/ijo.2015.42 26008137

[19] Nagin DS, Odgers CL. Group-based trajectory modeling in clinical research. Annu Rev Clin Psychol. 2010;6:109–38. doi: 10.1146/annurev.clinpsy.121208.131413 20192788

[20] Nagin DS. Group-based trajectory modeling: An overview. Ann Nutr Metab. 2014;65(2–3):205–10. doi: 10.1159/000360229 25413659

[21] Svefors P, Rahman A, Ekström E-C, Khan AI, Lindström E, Persson LÅ, et al. Stunted at 10 Years. Linear Growth Trajectories and Stunting from Birth to Pre-Adolescence in a Rural Bangladeshi Cohort. PLoS One. 2016;11(3):e0149700. doi: 10.1371/journal.pone.0149700 26934484 PMC4775024

[22] Faye CM, Fonn S, Levin J, Kimani-Murage E. Analysing child linear growth trajectories among under-5 children in two Nairobi informal settlements. Public Health Nutr. 2019;22(11):2001–11. doi: 10.1017/S1368980019000491 30940271 PMC6570617

[23] Investigators MEN. The Malnutrition and Enteric Disease Study (MAL-ED): Understanding the Consequences for Child Health and Development. Clinical Infectious Diseases. 2014;59:S193-330.

[24] Organization WH. WHO child growth standards: length/height-for-age, weight-for-age, weight-for-length, weight-for-height and body mass index-for-age: methods and development. World Health Organization. 2006.

[25] Caulfield LE, Bose A, Chandyo RK, Nesamvuni C, de Moraes ML, Turab A, et al. Infant feeding practices, dietary adequacy, and micronutrient status measures in the MAL-ED study. Clin Infect Dis. 2014;59 Suppl 4(Suppl 4):S248-54. doi: 10.1093/cid/ciu421 25305294 PMC4204612

[26] Psaki SR, Seidman JC, Miller M, Gottlieb M, Bhutta ZA, Ahmed T, et al. Measuring socioeconomic status in multicountry studies: Results from the eight-country MAL-ED study. Popul Health Metr. 2014;12(1):8. doi: 10.1186/1478-7954-12-8 24656134 PMC4234146

[27] Nagin DS. Analyzing developmental trajectories: A semiparametric, group-based approach. Psychological Methods. 1999;4(2):139–57. doi: 10.1037/1082-989x.4.2.139 11285809

[28] Nagin DS, Tremblay RE. Analyzing developmental trajectories of distinct but related behaviors: A group-based method. Psychol Methods. 2001;6(1):18–34. doi: 10.1037/1082-989x.6.1.18 11285809

[29] Nagin DS. Group-based modeling of development. Harvard University Press. 2005.

[30] Jones BL, Nagin DS, Roeder K. A SAS procedure based on mixture models for estimating developmental trajectories. Sociological Methods & Research. 2001;29(3):374–93. doi: 10.1177/0049124101029003005

[31] Andruff H, Carraro N, Thompson A, Gaudreau P, Louvet B. Latent class growth modelling: a tutorial. Tutorials in quantitative methods for psychology. 2009;5(1):11–24.

[32] van de Schoot R, Sijbrandij M, Winter SD, Depaoli S, Vermunt JK. The GRoLTS-Checklist: Guidelines for reporting on latent trajectory studies. Structural Equation Modeling: A Multidisciplinary Journal. 2016;24(3):451–67. doi: 10.1080/10705511.2016.1247646

[33] Breslow NE, Clayton DG. Approximate inference in generalized linear mixed models. Journal of the American Statistical Association. 1993;88(421):9–25. doi: 10.1080/01621459.1993.10594284

[34] Jones BL, Nagin DS. A note on a stata plugin for estimating group-based trajectory models. Sociological Methods & Research. 2013;42(4):608–13. doi: 10.1177/0049124113503141

[35] Namirembe G, Ghosh S, Ausman LM, Shrestha R, Zaharia S, Bashaasha B, et al. Child stunting starts in utero: Growth trajectories and determinants in Ugandan infants. Matern Child Nutr. 2022;18(3):e13359. doi: 10.1111/mcn.13359 35488408 PMC9218325

[36] Gough EK, Moodie EE, Prendergast AJ, Ntozini R, Moulton LH, Humphrey JH, et al. Linear growth trajectories in Zimbabwean infants. Am J Clin Nutr. 2016;104(6):1616–27. doi: 10.3945/ajcn.116.133538 27806980 PMC5118730

[37] Krebs NF, Hambidge KM, Westcott JL, Garcés AL, Figueroa L, Tshefu AK, et al. Birth length is the strongest predictor of linear growth status and stunting in the first 2 years of life after a preconception maternal nutrition intervention: The children of the Women First trial. Am J Clin Nutr. 2022;116(1):86–96. doi: 10.1093/ajcn/nqac051 35681255 PMC9257468

[38] Hoddinott J, Behrman JR, Maluccio JA, Melgar P, Quisumbing AR, Ramirez-Zea M, et al. Adult consequences of growth failure in early childhood. Am J Clin Nutr. 2013;98(5):1170–8. doi: 10.3945/ajcn.113.064584 24004889 PMC3798075

[39] Walker SP, Grantham-Mcgregor SM, Powell CA, Chang SM. Effects of growth restriction in early childhood on growth, IQ, and cognition at age 11 to 12 years and the benefits of nutritional supplementation and psychosocial stimulation. J Pediatr. 2000;137(1):36–41. doi: 10.1067/mpd.2000.106227 10891819

[40] Schwarzenberg SJ, Georgieff MK, Committee on Nutrition. Advocacy for improving nutrition in the first 1000 days to support childhood development and adult health. Pediatrics. 2018;141(2):e20173716. doi: 10.1542/peds.2017-3716 29358479

[41] Derbyshire E, Obeid R. Choline, neurological development and brain function: A systematic review focusing on the first 1000 days. Nutrients. 2020;12(6):1731. doi: 10.3390/nu12061731 32531929 PMC7352907

[42] Roth DE, Krishna A, Leung M, Shi J, Bassani DG, Barros AJD. Early childhood linear growth faltering in low-income and middle-income countries as a whole-population condition: Analysis of 179 Demographic and Health Surveys from 64 countries (1993-2015). Lancet Glob Health. 2017;5(12):e1249–57. doi: 10.1016/S2214-109X(17)30418-7 29132614 PMC5695758

[43] Bhutta ZA, Akseer N, Keats EC, Vaivada T, Baker S, Horton SE, et al. How countries can reduce child stunting at scale: Lessons from exemplar countries. Am J Clin Nutr. 2020;112(Suppl 2):894S-904S. doi: 10.1093/ajcn/nqaa153 32692800 PMC7487427

[44] Wells JCK, Briend A, Boyd EM, Berkely JA, Hall A, Isanaka S, et al. Beyond wasted and stunted-a major shift to fight child undernutrition. Lancet Child Adolesc Health. 2019;3(11):831–4. doi: 10.1016/S2352-4642(19)30244-5 31521500

