# Supplementary material for: Linear growth trajectories in the first two years of life predict attained linear growth and stunting at five years: Results from the MAL-ED multi-country birth cohort study
Source: PLoS One. 2026 Apr 6;21(4):e0329596. doi: 10.1371/journal.pone.0329596 (PMC13052894; doi:10.1371/journal.pone.0329596)
Supplement: S1 Table — (DOCX) [file pone.0329596.s001.docx]

**S1 Table. Association of LAZ trajectories during the first two years of life with height-for-age z-score (HAZ) at age 60 months (n = 1047): full adjusted model**

| **Variables** | **Height-for-age z score (HAZ)** | |
| --- | --- | --- |
|  | **β (95% CI)** | **p-value** |
| LAZ trajectory group |  |  |
| Class 1: Severely attenuated | -2.10 (-2.26, -1.94) | <0.001 |
| Class 2: Moderately attenuated | -1.34 (-1.45, -1.23) | <0.001 |
| Class 3: Mildly attenuated | -0.71 (-0.81, -0.60) | <0.001 |
| Class 4: Stable | Reference |  |
| Class 5: Improved | 0.86 (0.67, 1.04) | <0.001 |
| Child sex |  |  |
| Male | Reference |  |
| Female | -0.17 (-0.24, -0.09) | <0.001 |
| Daily protein intake in grams | 0.008 (0.004, 0.012) | <0.001 |
| Daily fat intake in grams | -0.002 (-0.006, 0.001) | 0.148 |
| Daily carbohydrate intake in grams | -0.0001 (-0.0008, 0.0006) | 0.805 |
| WAMI index | 0.58 (0.30, 0.86) | <0.001 |
