# Supplementary material for: Linear growth trajectories in the first two years of life predict attained linear growth and stunting at five years: Results from the MAL-ED multi-country birth cohort study
Source: PLoS One. 2026 Apr 6;21(4):e0329596. doi: 10.1371/journal.pone.0329596 (PMC13052894; doi:10.1371/journal.pone.0329596)
Supplement: S2 Table — (DOCX) [file pone.0329596.s002.docx]

**S2 Table. Association of LAZ trajectories during the first two years of life with stunting at age 60 months (n = 1047): full adjusted model**

| **Variables** | **Stunting** | |
| --- | --- | --- |
|  | **OR (95% CI)** | **p-value** |
| LAZ trajectory group |  |  |
| Severely attenuated (Class 1) | 132.78 (61.58, 286.52) | <0.001 |
| Moderately attenuated (Class 2) | 16.00 (10.28, 24.91) | <0.001 |
| Mildly attenuated or Stable or Improved (Class 3 or 4 or 5) | Reference |  |
| Child sex |  |  |
| Male | Reference |  |
| Female | 1.23 (0.84, 1.81) | 0.291 |
| Daily protein intake in grams | 0.98 (0.96, 1.00) | 0.123 |
| Daily fat intake in grams | 1.01 (0.99, 1.02) | 0.373 |
| Daily carbohydrate intake in grams | 0.999 (0.996, 1.003) | 0.943 |
| WAMI index | 0.24 (0.07, 0.76) | 0.004 |
